# NVIS Multicarrier Modulations for Remote-Sensor Applications

**DOI:** 10.3390/s20216232

**Published:** 2020-10-31

**Authors:** Josep M. Maso, Tomas Gonzalez, Jordi Male, Joaquim Porte, Joan L. Pijoan, David Badia

**Affiliations:** La Salle Campus, Ramon Llull University, 08022 Barcelona, Spain; josep.maso@salle.url.edu (J.M.M.); tomas.gonzalez@salle.url.edu (T.G.); jordi.male@salle.url.edu (J.M.); joaquim.porte@salle.url.edu (J.P.); david.badia@salle.url.edu (D.B.)

**Keywords:** Remote Sensing, HF, NVIS, USN, OFDM, IoT, STANAG, MIL-STD 188 110 D

## Abstract

The number of Internet of Things (IoT) devices has experienced a large growth during the last decade, as well as the data volume gathered from remote sensors. Satellites are still a suitable communication method and may be preferable for a remote ubiquitous sensor network (USN), which sometimes are located in places without much communications infrastructure where coverage is the principal drawback. Alternatively, the proposed solution for this article aims at a near-vertical incidence skywave (NVIS) channel for high frequencies (HF) with a low-cost platform, allowing a low-power transmissions coverage area up to 250 km for USN. The HF standards are focused on generic communication channels not being robust for NVIS communications. In this article we study and test an alternative based on orthogonal frequency-division multiplexing (OFDM) modulations to make them more robust and less dependent on the channel NVIS communications. For that purpose, we test the HF standard modulations and a designed OFDM modulation to prove the robustness of each. This study has been tested between Barcelona and Tarragona, using different transmission power levels and modulation orders.

## 1. Introduction

It is not news that the number of sensors and mobile devices is increasing enormously every day in the current world. The infrastructure of the communications for these devices is very extended in areas with a high population. However, some areas in the world do not have such infrastructure due to complex orography, which makes communications between the transmitter and receptor almost impossible. Normally, the most extended way to communicate in these places is the use of satellite services, which do not need any terrestrial network infrastructure. Nowadays, the environmental impact of satellite deployments [1] and their high cost has made researchers discover new methods of communications especially with the aim of collecting data through remote sensors for several scientific studies.

Remote sensing became an extended study focus making use of new technologies such as light detection and ranging (LIDAR), artificial intelligence (AI) [2], machine learning [3], geocoding algorithms [4], deep convolutional neural networks [5] or multi-sensor fusion positioning [6], being part of some examples of the wide range of technologies that sensing uses.

As an alternative to satellite communications, the use of ionospheric reflection has been under study for several years even in scenarios such as Antarctica where there is almost no kind of infrastructure, and the deployment of network communications are practically unfeasible due to its complicated terrain [7]. Near-vertical incidence skywave (NVIS) offers an alternative solution in order to collect data from remote sensors. NVIS consists in transmitting a signal which near vertically rebounds in the ionosphere for frequencies under 10 MHz (high frequency) giving 250 km area coverage with low-cost equipment and an easy deployment system [8] in comparison to satellite communications.

To maintain a standard for NVIS communications, high-frequency (HF) frame protocols MIL-STD 188 110C Appendix D [9] and STANAG [10] are used. These frame protocols are well designed but the results for shorter distances are not borne in mind setting aside significant effects that degrade the signal as multipath which are critical in the mornings and evenings because of the use of narrowband modulations. In scenarios with big multipath, the equalization becomes a very complex work. To avoid this effect, orthogonal frequency-division multiplexing (OFDM) modulation can bring a solution due to its characteristics of being very robust against the multipath and the equalization being very simple to implement in real time.

In this paper we present an alternative to the standards by the use of a wideband OFDM modulation for NVIS based on a low-cost and low-power platform for remote sensors, which can be a solution to multipath. This solution aims to improve NVIS communications and make a more robust system. To achieve this solution, this study has two principal goals: the proposal of a new ionospheric transmission configuration and to have the ideal trade-off between narrow-band modulations and OFDM in terms of power consumption for low-power systems. If we take into account the possible applications, the starting point is the battery consumption for remote sensors. For that reason, the input back off (IBO) plays an essential role in the OFDM modulation. This parameter lets us increase the average power cutting the peaks that the OFDM produces due to the subcarriers division and is more adequate for the ubiquity for portable sensors.

Moreover, to improve NVIS, the use of two different waves, known as the ordinary and extraordinary waves, can benefit the communication. These waves are caused birefringence nature of the ionospheric plasma in the presence of the geomagnetic field [11]. Also, these two characteristic waves affect the radio wave differently, creating two decorrelated channels. This decorrelation opens new horizons in our link, as polarization diversity appears as an improvement for the NVIS link when used combined with multiple antennas in reception. Therefore, this paper presents the application of polarization diversity in an NVIS single input multiple output (SIMO) scenario.

This article is organized as follows. In Section 2, we explain the developed low-cost system, including hardware, software, the OFDM design and the diversity technique to overcome results. In Section 3, we explain the test’s design and the frame protocols performed. In Section 4, the comparison between modulations based on the results are shown. Finally, Section 5 gives the conclusions.

## 2. System Description

This section introduces the basis of the NVIS characteristics and the leading technologies present in the developed platform used to achieve the study, as different techniques are used in the optimization of the platform. Additionally, an overall vision of the components and software are explained.

### 2.1. Near-Vertical Incidence Skywave (NVIS)

NVIS propagation consists of the transmission of HF electromagnetic waves between 3 to 10 MHz with an angle above 70° to the ionosphere which can make this signal rebound and reach distances up to 250 km [8]. The rebounds of the waves are possible due to the solar radiation and the terrestrial magnetic field. The reflection depends on the ionization of the ionospheric layers and is strongly dependent on the frequency and solar activity. The achieved bit rates are not high, but enough for Internet of Things (IoT) devices, so NVIS can be a good alternative for a ubiquitous sensor network (USN), having a low cost due to the use of software defined radio (SDR) [5].

The main challenge of this kind of communications is the availability of the rebounds. This fact relies on the ionosphere layers, which are D, E and F. D appears during the day but prevents the rebounds under 10 MHz and attenuates the signal. The E layer is the first which allows the HF signals to rebound followed by the F layer, which is divided into F1 and F2. Both are present during the day, but during the night the F1 disappears. The F2 due to its stability is the layer with which the test transmissions have been performed.

Lastly, the ionosphere with NVIS has some difficulties in the design because of the channel effects produced by the ionosphere rebounds as studied by Vilella [11], Jodalen [12], Hervas [13], and Cannon [14] but the proposed NVIS protocol overcomes that.

Also, this protocol has a SIMO technique which overcomes results that sustain the study of different polarization ways in order to receive the NVIS signal studied by Erhel [15]. In our case, the polarization diversity is used to make the modulation performance better.

A preliminary experimental approach of the noise level in channels between 3 and 24 KHz was studied by Bechet, Bechet and Miclaus [16], but the proposed solution takes the channel and noise estimation by the PN sequence which does a channel profile. Also, the equalization with this sequence became more efficient.

### 2.2. Overview of the System

The current platform allows a continuous transmission with a radius of 250 km because of the NVIS channel. Talking about costs and pollution, the developed NVIS platform is affordable compared to any satellite.

**Figure 1:**
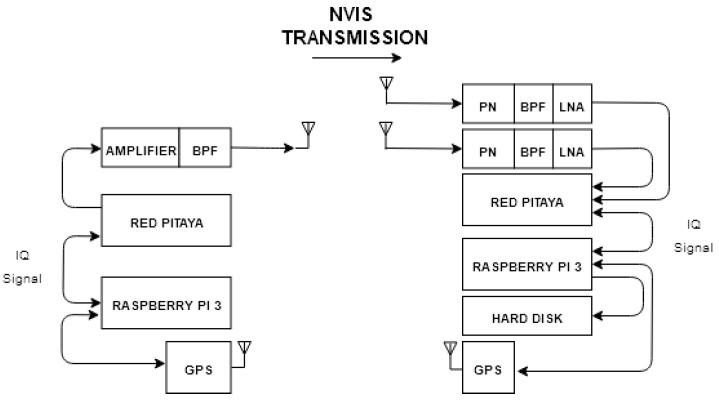
Near-vertical incidence skywave (NVIS) transmission scheme (transmitter in the left and receiver in the right).

The system description is explained below, taking into account all different parts, and finally, a graphical scheme is shown in Figure 1. Mainly, the Red Pitaya is the core of the system, and is in charge of all high-speed RF signal-processing. In the other hand, the Raspberry Pi 3 is in charge of all base-band signal-processing.

- Software-Defined Radio (SDR)/Red Pitaya

SDR is key to the development of the platform [17] due to its scalability and is done through two Red Pitaya STEMLab 125-14 that contains field-programmable gate array (FPGA) Xilinx Zynq 7010 System on Chip (SoC). The low-cost Red Pitaya platform have two analogic digital converters (ADC) and two digital analogic converters (DAC) of 14 bit resolution allowing the transmission and reception of both ordinary and extraordinary waves. The SDR methodology, some settings such as the carrier frequency, bandwidth and modulation can be changed in a very dynamic way.

- Raspberry Pi 3

The time synchronization between transmitter and receiver is performed with GPS controlled as a peripheral from Raspberries Pi 3 [18]. In the transmitter side, the Raspberry has all the transmission test signal files to be transmitted. All transmission signal files are sent to the Red Pitaya via Ethernet and transmitted when needed. For the reception, the process just explained works backwards. The Red Pitaya send the data received to the Raspberry, core that will gather all the information obtained in order to process it with data-processing software [19].

- Amplifier and Low Noise Amplifier (LNA)

For our lab tests, an amplifier of 48.5 dB is used in order to reach 50 dBm signal transmissions. Bonn BLWA 0103-250 class A is the chosen model, which can works correctly between 1.5 and 30 MHz [20]. In contrast, at the receiver side, the signal has to be preamplified to 30 dB by an LNA for a proper demodulation. In the final system, the amplifiers used are different, being more affordable.

- Filter

A band pass filter (BPF) is needed to limit the frequencies used for NVIS channel (3–10 MHz). Due to strong interferences in that band, we have used a 2 MHz BPF centered on 5.4 MHz to avoid the saturation of the ADC at the reception.

- Antenna

Our system needs to be easily deployed, and the election of the antennas is intended for this purpose as far as possible. The chosen antennas are an inverted V which have a gain of 6.8 dBi [21], with a simple installation using one single mast. A total of three identical antennas are needed in our scenario, all tuned to frequency of 5 MHz calculated from the revised ionograms of Observatori del Ebre [22].

**Figure 2:**
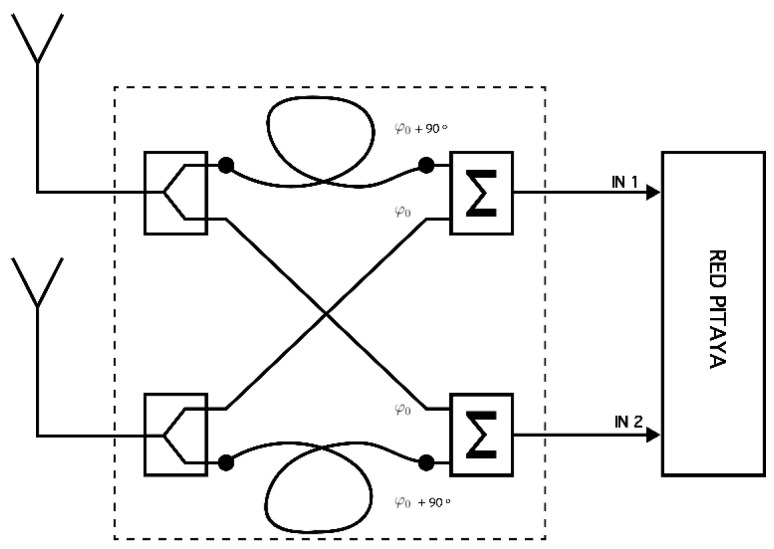
Phasing box of the receiver system.

One of the three antennas is placed in the transmitter, while the other two are set perpendicularly at the receiver with the purpose of receiving both right-handed circular polarization and left-handed circular polarization waves simultaneously. To receive both waves, the antenna configuration on the receiving requires a phasing network as we can see in Figure 2.

The aforementioned phasing box was developed in order to perform the delays explained using coaxial cables. Each of the antenna inputs is divided into two identical signals with a radio frequency splitter, generating 4 signals (2 for each antenna). The paths followed by each antenna are identical. One of the cables is lengthened with a quarter wave phasing line, providing a 90° phase shift, while the other signal is connected directly to an RF combiner. This process is repeated identically for both antenna wires, resulting in an output of two dipole antennas with a phase difference of either +90° or −90° between each other. Equation (1) shows the unification of the two antennas which includes the non-phased wave in (2) and the 90° phased wave in (3). It is important to mention that this system has been designed for a unique frequency. For wideband studies, the phasing network should be frequency adaptive and implemented in the FPGA.

(1) Ē(z,t) = Ēx(z,t) + Ēy(z,t)

(2)  Ēx(z,t) = Ēo · cos(wt − kz) · x 

(3) $$\;Ēy(z,t)\;=\;Ēo\;\cdot \;\cos(\mathrm{wt}\;-\;\mathrm{kz})\;+\;\frac{\pi }{2}\cdot y\;\;$$

### 2.3. Orthogonal Frequency-Division Multiplexing (OFDM)

The narrow-band modulations (PSK, FSK and QAM) studied in previous works are good enough for remote-sensing applications, but in low-multipath scenarios. Our OFDM proposal is suited to manage strong multipath with easy equalization methods. The study of this modulation lies in its capacity to avoid the multipath effect and the easy way to equalize it. It is known that in ionospheric communications the evening transmissions are plenty of multipath compared to the morning, being the OFDM an excellent option to avoid those effects [23].

The spectral efficiency is a good characteristic as well as the computational efficiency due to the FFT and IFFT. The modulation process is done by the IFFT of all the M-QAM or M-PSK symbols, which form the OFDM symbols. Equation (4) describes the IFFT process of the symbols where Nsc is the number of subcarriers and Sk is the modulated symbols in QAM/PSK. This process ensures data transmission in multiple parallel subtransmissions at lower speed, but in a robust way, which helps the stability of any communication system.

(4) $$x\left[n\right]=\frac{1}{\sqrt{\mathrm{Nsc}}}\overset{k=\mathrm{Nsc}-1}{\underset{k=0}{\sum }}{\mathrm{Sk}\cdot e}^{\frac{{j2\pi n}^{k}}{\mathrm{Nsc}}},\;0\;\leq \;n\;\leq \;\mathrm{Nsc}$$

As a drawback, the OFDM peak consumption is higher than any other narrow-band modulations because of the subcarrier division creates peaks having as a result a high peak to average power ratio (PAPR) which is the difference between the peak power and the average power.

The configuration of the OFDM to be transmitted in our tests is based to be the most similar as the HF standards to maintain the most similar comparison. The design of the OFDM configuration requires a previous study of the channel to define every parameter. This channel analysis and definition of the OFDM configuration for high multipath is defined in a previous article [24]. Taking account the designed OFDM, first of all, bandwidth of the OFDM signal is 3 KHz as the HF standards. The symbol length was calculated first with a value of 9.33 ms as in [24] to set the number of subcarriers. As a result of that, 28 subcarriers will be transmitted being one of them a DC null to avoid offset effects. The configuration designed makes every subcarrier to be about 107 Hz. Every frame packet is composed of 7 OFDM symbols with a duration of 86.31 ms, which are obtained due to the coherence time (10 s) [24]. One of the OFDM symbols is a pilot to estimate the channel to perform the zero-forcing equalization which is calculated as we can see in (6). The zero-forcing consists in applying the inverse of the estimated channel calculated with the pilot symbol. In (5) is shown a simple equation of a transmitted signal in which Y(f) means the signal affected by a channel, the X(f) refers to the raw signal and the H(f) means the channel response. The received pilot is compared to the transmitted one to take a value of how the channel changes and the OFDM symbols are multiplied by the inverse of the received channel response as in (6).

(5) Y(f) = H(f) · X(f)

(6) $$\;C(f)\;=\;\frac{1}{Hest\left(f\right)}\;$$

The delay spread is a key measure of the multipath received, being the time between the first and the last path received. In [24] this was already calculated for an NVIS channel, and its value is 2.75 ms, for that reason the cyclic prefix is calculated in relation to this value, adding a small leeway. Finally, 3 ms of copied useful data inserted at the beginning of the OFDM symbol (CP) avoid the interferences between neighbor symbols produced by the multipath of the channel. The application of this technique is the reason of why the OFDM is a good option to avoid the multipath intersymbol interference (ISI).

One of the weak points of the OFDM is the high PAPR, as mentioned, the OFDM modulation produces high peaks which reduce the average power of the modulation. Also, this is reduced by the IBO application to crop the peaks and then rising the average power. In [25] the IBO performance was analyzed, and it was concluded that the lowest values of IBO produce high in-band distortion that degrades the EVM, whereas the high values of IBO reduce the mean transmitted power.

The initial design is done with an IBO of 3 dB because the average power was too small compared to the narrow-band modulations. After doing the first comparison, more IBO values (4.5, 6, 7.5 and 9 dB) are studied to make the OFDM more efficient. The bits in use are calculated by multiplying the bits/symbol, the number of data OFDM symbols and the number of data subcarriers. Finally, the summarized configuration of the OFDM is shown in Table 1.

### 2.4. Polarization Diversity

Diversity techniques are being applied in many communication fields. Spatial diversity, frequency diversity and time diversity are methods applied in many frequency bands and scenarios. Polarization diversity is a diversity mode that may be applied in very specific environments and is based on two different channels with particular properties.

The ionosphere is an ionized layer of the atmosphere due to solar radiation. Its electrons vibrate at the frequency of the incoming waves, acting as small dipoles. These vibrations are usually elliptical in shape and occur in both directions. Because of this, the ionosphere creates two opposite channels due to the movement of the electrons. These channels are completely decorrelated and change the polarization of the wave to a circular one, even if the emitted signal is linearly polarized [26].

When having two isolated channels, multiple input multiple output (MIMO) appears as a method to exploit their multipath propagation. This work focuses on the ionospheric channel and the development of a SIMO system that benefits from the two characteristic waves, the ordinary and the extraordinary waves. Both MIMO and SIMO are valid solutions, with the MIMO being the one that can provide better results as more antennas are involved in the scenario and more techniques can be applied (space-time coding, for example). A SIMO scenario [27] demonstrated that the selection-combining (SC) technique helps to reach better results in terms of E_b_/N_0_.

Despite these gains, the HF antennas are too big and most of the times it is necessary to install a mast. The use of diversity-polarization makes sense in the receiver side because it can help to minimize the power consumption and the size of the transmitter antenna. This article studies the SC and equal gain combining (EGC) techniques to outperform results. SC compares the two different signals received (two different channels) in terms of E_b_/N_0_ and then ignores the worse result. On the other hand, EGC makes the coherent sum of both channels to get an increase of the bit energy [27].

Geoscience and the complete understanding of how the atmosphere’s layers work take a leading role in the development and implementation of telecommunication’s applications. Remote sensing, for instance, can directly benefit from the ionosphere’s studies by the application of techniques based on the ionospheric properties. This paper shows, for example the usage of polarization diversity as an improvement of a remote-sensing network.

## 3. Tests

In this section, we explain the area where we tested the different transmissions with an NVIS channel and the organization of the tests to be transmitted.

There were two different tests: the first one was dedicated to a simple comparison between the narrow-band and the multicarrier-band modulation to know the viability and efficiency respect narrow-band modulations, and the second one was to optimize the OFDM transmitted making use of the IBO which helps to find a power transmission for a low-consumption system which means smaller batteries.

### 3.1. Test Area

**Figure 3:**
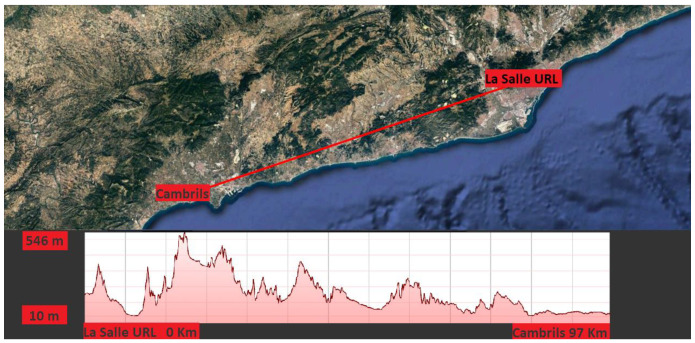
Barcelona-Cambrils link.

In La Salle University URL (Barcelona, Spain) there is an inverted V antenna acting as a transmitter NVIS node. The receptor is approximately 97 km away (Cambrils, Tarragona) where La Salle has a specific lab with the same antenna but making use of SIMO technique, so there are two of them and the phasing box. Figure 3 shows the link established within the line of sight between the transmitter and the receiver, as we can see at the profile elevation with an elevation peak of 546 m.

### 3.2. Frame Protocol and Tests Design

The tests follow a 10-minute plan, in which the first 5 min the platform does not transmit, and throughout the next 5 min, we increase the order of modulation for each modulation (from 2 to 32) after each minute. This process is repeated six times during one hour but increasing the transmission power by 3 dBs from 35 dBm to 50 dBm every 10 min. The summary is shown in Table 2.

Each transmission includes a 6th PN sequence with a resampling of 8 and 5 ms length as in [24] that is used to synchronize the demodulation of the frames. The PN sequence has been designed not to be affected by the delay spread and Doppler shift. A single-tone of 600 Hz of 60 ms length is used to correct the Doppler shift caused by the inaccuracy of the clocks of the Red Pitaya which generates a maximum Doppler of 17.5 Hz [24], higher than the ionospheric channel shift which values under 10 Hz. Taking into account a tone of 600 Hz in the worst case will be of 580 Hz due to the Doppler shift. To assure the measure, if we consider a 550 Hz received tone, the measure of it to correct the Doppler shift effect will be of 33 cycles. In the case of using a DC tone, for measuring 1 Hz of Doppler shift, the measure would consider only a 16th part of a cycle (60 ms), which is not enough for an accurate result. The entire tests have a fixed bandwidth of 3 kHz and a frequency of 5.4 MHz.

**Figure 4:**
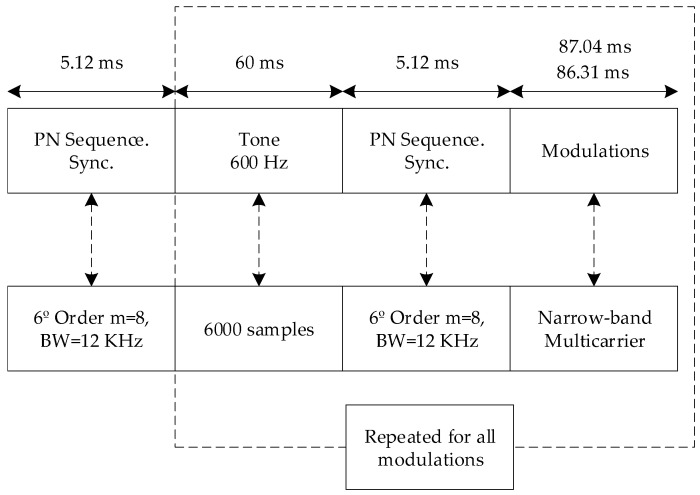
Frame design for each modulation.

Each transmission contains 200 packets (50 for each modulation) of 162 symbols with a resample of 34 to achieve 3 KHz of channel bandwidth. In the Figure 4 below, the frame design is outlined. Each test transmission is composed of a frame that includes a PN sequence to synchronize each transmission, and then there are a single-tone and a PN sequence for every modulation packet. To maintain the time standards of each packet with the OFDM modulation, the narrow-band modulations are 87.04 ms in length and the multicarrier modulation is 86.31 ms in length. Once the packet is transmitted this process is repeated for the rest of the packets.

A good point of study is the optimization of the average power of the OFDM due to the high peaks produced by the multiple subcarriers. This optimization allows the platform to have greater autonomy or smaller batteries to be integrated in low-power applications. Numerous techniques overcome the results of the OFDM bit error rate (BER) due to the increase of the average power. However, we opted for the IBO study.

The initial tests start with IBO = 3 dBs just to reduce the principal peaks and compared directly with narrow-band modulations. After that, there is a specific IBO sweep to obtain the optimal one (same structure as before), which also helps the reduction of power consumption indirectly. Is true that high values of IBO increase the average power (energy) and overcome the bit error, but thanks to that, the peak power could be decreased.

## 4. Results

The most relevant results obtained from the test performed will be shown in this section. We will analyze the BER obtained depending on the E_b_/N_0_, the cumulative distribution function (CDF) of BER for a specific E_b_/N_0_, the PAPR, average power and peak power obtained depending on the IBO, the CDF BER depending on the IBO and the improvement of the communication by using polarization techniques at the receiver system. These tests took around two weeks transmitting and receiving a total amount of 28 MB of data.

### 4.1. BER vs. E_b_N_0_

At first, we will analyze the BER obtained depending on the E_b_/N_0_ for each transmitted modulation and modulation order symbolized with M. These results show us the robustness of each modulation in front of the NVIS channel. The results are better when the line takes lower values.

#### 4.1.1. BER vs. E_b_/N_0_ M = 4

**Figure 5:**
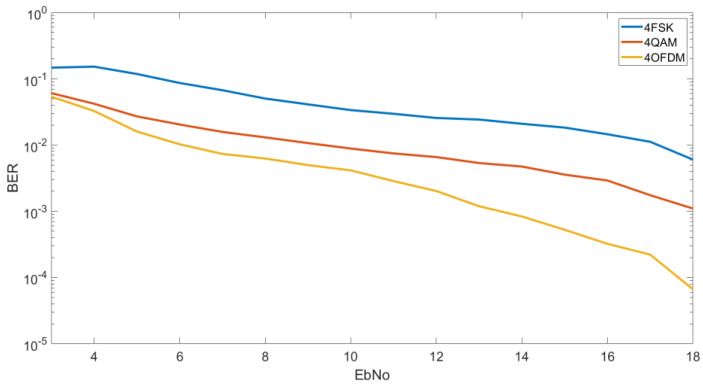
BER vs. E_b_/N_0_ M = 4.

In Figure 5, we can see the results obtained for the 4FSK, 4QAM and the OFDM designed with a 4QAM modulation. As we can see in the graphic, the OFDM is the most robust modulation to be transmitted. In the best case, for an E_b_/N_0_ of 18 dB we can obtain a BER of 6 × 10^−5^ by using an OFDM modulation, a BER of 10^−3^ by using a 4QAM and a BER of 6 × 10^−3^ by using a 4FSK. For a lower E_b_/N_0_ as 10 dB, we can see that the results are more similar with a BER of 4 × 10^−3^ for the OFDM, a BER of 8 × 10^−3^ for the 4QAM and a BER of 3 × 10^−2^ for the 4FSK.

#### 4.1.2. BER vs. E_b_/N_0_ M = 8

**Figure 6:**
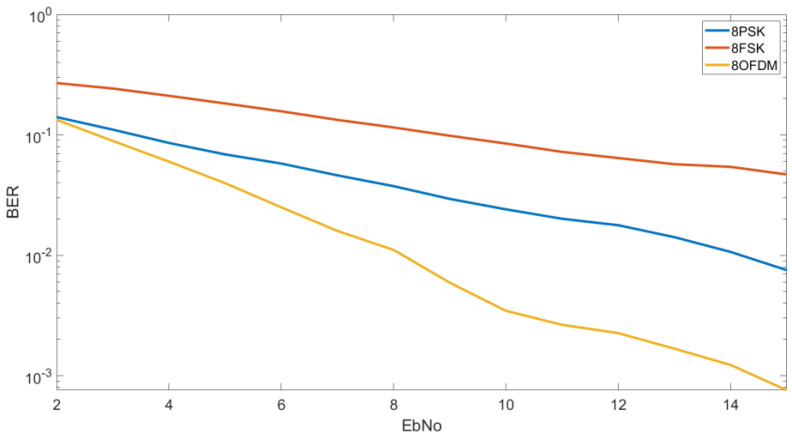
BER vs. E_b_/N_0_ M = 8.

In Figure 6, we can see the results obtained for the 8FSK, 8QAM and the OFDM designed with an 8QAM modulation. In this case the OFDM is shown again to be the most robust. For an E_b_/N_0_ of 10 dB we can obtain a BER of 4 × 10^−3^ by using an OFDM modulation, a BER of 2 × 10^−3^ by using an 8QAM and a BER of 8 × 10^−1^ by using an 8FSK. For a lower E_b_/N_0_ as 5 dB, we can see that we obtain a BER of 4 × 10^−2^ for the OFDM, a BER of 7 × 10^−2^ for the 8QAM and a BER of 2 × 10^−1^ for the 8FSK.

#### 4.1.3. BER vs. E_b_/N_0_ M = 16

**Figure 7:**
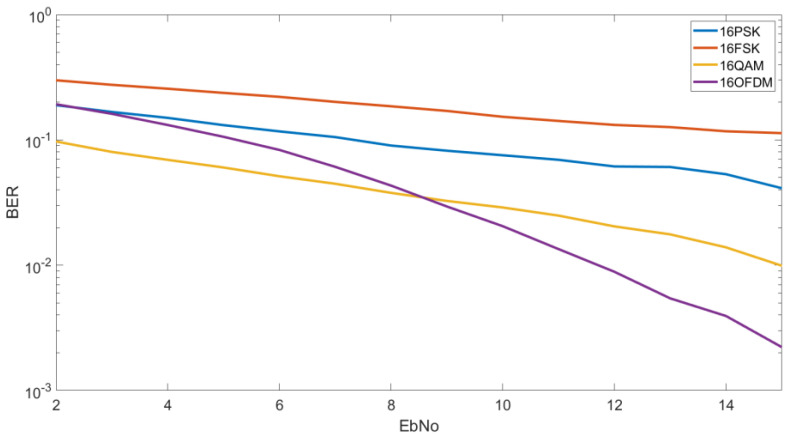
BER vs. E_b_/N_0_ M = 16.

In Figure 7, we analyze the results of 16FSK, 16QAM, 16PSK and the OFDM designed with a 16QAM modulation. In this case, OFDM is only the most robust modulation for high E_b_/N_0_ and 16QAM the most robust modulation for low E_b_/N_0_. As we can see for an E_b_/N_0_ of 10 dB we can obtain a BER of 2 × 10^−2^ by using an OFDM modulation, a BER of 3 × 10^−3^ by using a 16QAM, a BER of 7 × 10^−2^ by using an 8PSK and a BER of 2 × 10^−1^ by using a 16FSK. For lower E_b_/N_0_ as 5 dB, we can see that we obtain a BER of 6 × 10^−2^ for the 16QAM, a BER of 10^−1^ by using an OFDM, a BER of 10^−1^ for the 16PSK and a BER of 2 × 10^−1^ for the 16FSK.

### 4.2. BER Cumulative Distribution Function (CDF)

Once analyzed the BER depending on the E_b_/N_0_ it is important to analyze for each E_b_/N_0_ the CDF of the modulations depending on the order of modulation. By this test, we can obtain more information about the robustness of each modulation with low energy per bit and the probabilities of obtaining a low BER. All graphics of this section shows us in the Y-axis the probability of obtaining a BER lower than a value X_o_ represented on the X-axis. The results are better when the line is at the top left.

#### 4.2.1. BER vs. E_b_/N_0_ = 5 dB M = 4

**Figure 8:**
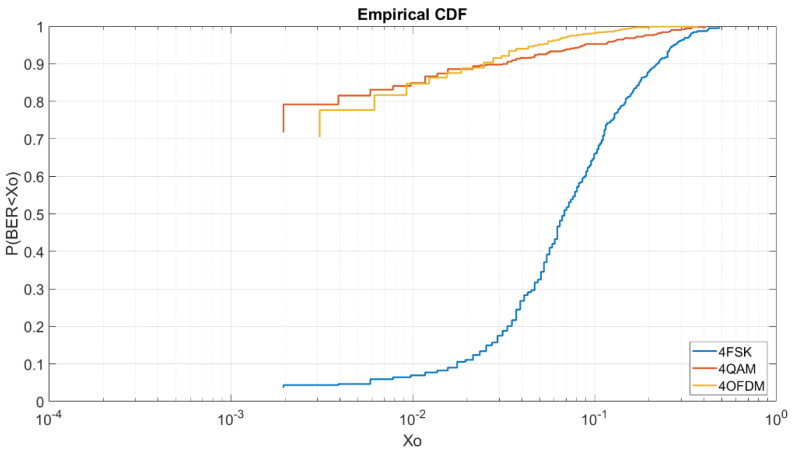
BER vs. E_b_/N_0_ = 5 dB M = 4.

In Figure 8, we can see for a low E_b_/N_0_ of 5 dB the behavior of each modulation and robustness. We can see that the OFDM and QAM have the best results in a very similar way. The 4QAM have a probability of 79% to obtain a BER lower than 2 × 10^−3^ and the OFDM have a probability of a 78% to obtain a BER lower than 3 × 10^−3^. The 4FSK as we can see is highly affected by the low energy bit transmission with a probability of a 4% to obtain a BER lower than 2 × 10^−3^. As we can see in this graphic, the OFDM is shown to be a little bit worse than the 4QAM even though in the graphic of Figure 5 it is shown to be more robust. As we can see at Figure 8 for higher BERs the OFDM is more robust than the QAM, for a BER lower than 10^−1^ we have a probability of 98% for the OFDM, a probability of 95% for the 4QAM and a probability of 64% for the 4FSK. For this reason, for a low E_b_/N_0_ of 5 dB, the OFDM obtains better results in terms of average but for transmission with the minimum errors it is better to use the 4QAM.

#### 4.2.2. BER vs. E_b_/N_0_ =5 dB M = 8

**Figure 9:**
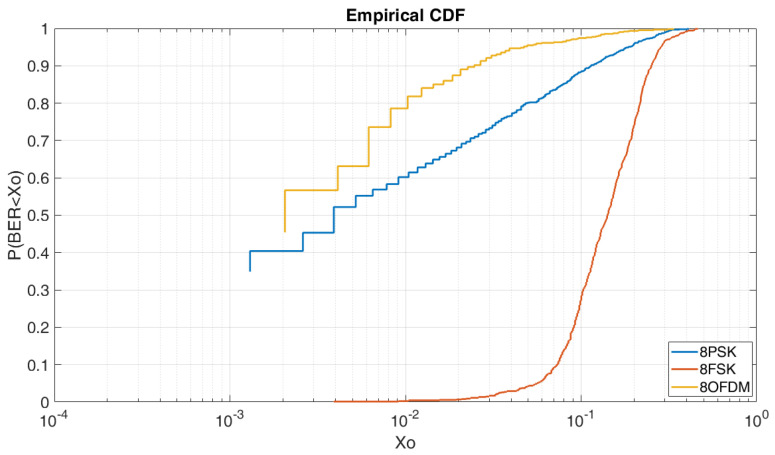
BER vs. E_b_/N_0_ = 5 dB M = 8.

In Figure 9, we can see the CDF for an E_b_/N_0_ of 5 dB and order of modulation 8. As we can see, in this case, the OFDM obtains the best results in comparison of the 8PSK and the 8FSK. To obtain a BER lower than 2 × 10^−3^ we have a probability of 56% for the OFDM and a probability of 45% for the 8PSK. For the 8FSK, we have a probability of a 1% to obtain a BER lower than 4 × 10^−3^.

#### 4.2.3. BER vs. E_b_/N_0_ = 5 dB M = 16

**Figure 10:**
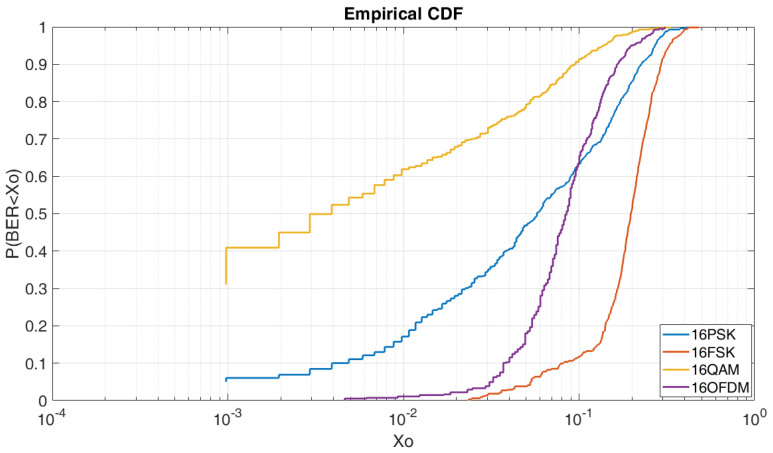
BER vs. E_b_/N_0_ = 5 dB M = 16.

In Figure 10, we can see the CDF for a E_b_/N_0_ of 5 dB and order of modulation of 16. In this case, contrary to Figure 9, 16QAM obtains the best results in comparison of the 16PSK, the 16FSK and the OFDM. As we can see, to obtain a BER lower than 5 × 10^−3^ we have a probability of 54% for the 16QAM, a probability of 10% for the 16PSK, and a probability of a 4% for the OFDM. At this figure, the OFDM is highly affected due to the increase of modulation order. Finally, in this case, the 16FSK has a probability of 10% to obtain a BER lower than 10^−1^.

#### 4.2.4. BER vs. E_b_/N_0_ = 8 dB M= 4

**Figure 11:**
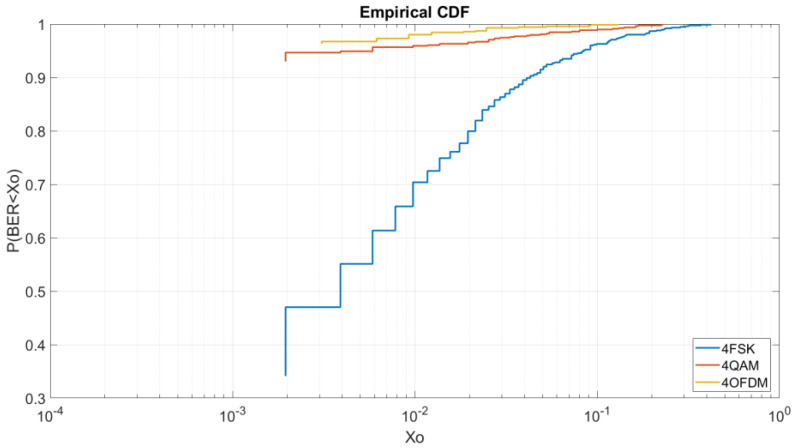
BER vs. E_b_/N_0_ = 8 dB M = 4.

Once the order modulations with a low E_b_/N_0_ of 5 dB are analyzed, we will analyze the same modulations with a E_b_/N_0_ of 8 dB. At Figure 11, we can see that the OFDM has better results than the 4QAM due to the increase of E_b_/N_0_. To obtain a BER lower than 6 × 10^−3^ we have a probability of 97% for the OFDM, a probability of 95% for the 4QAM and a probability of 61% for the 4FSK. For this E_b_/N_0,_ we can see in Figure 11 that the OFDM always has better results than the 4QAM unlike the CDF of Figure 8.

#### 4.2.5. BER vs. E_b_/N_0_ = 8 dB M = 8

**Figure 12:**
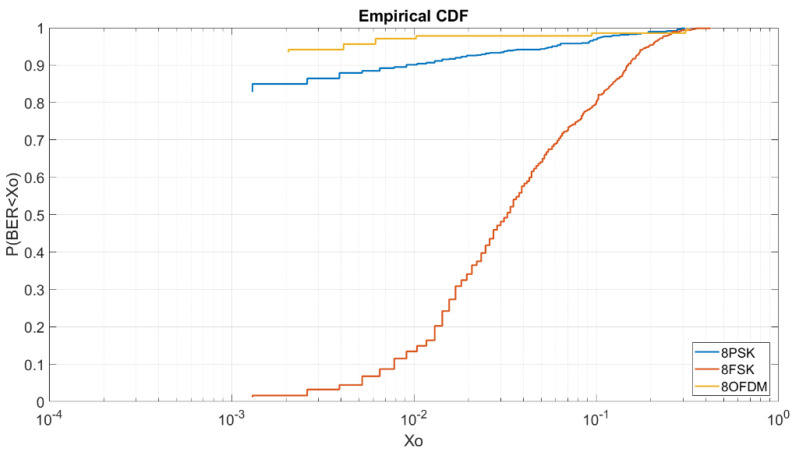
BER vs. E_b_/N_0_ = 8 dB M = 8.

At Figure 12, we can analyze again that the order 8 OFDM is more robust than the 8PSK and 8FSK. As we can see, to obtain a BER lower than 4 × 10^−3^ we have a probability of 95% for the OFDM, a probability of 88% for the 8PSK and a probability of 4% for the 8FSK.

#### 4.2.6. BER vs. E_b_/N_0_ = 8 dB M = 16

**Figure 13:**
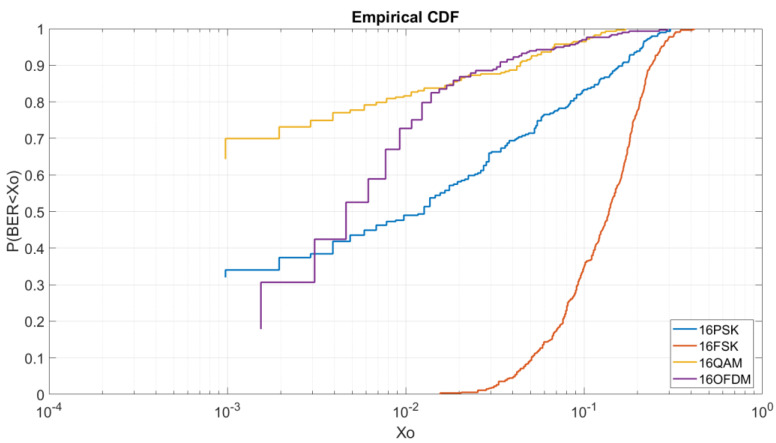
BER vs. E_b_/N_0_ = 8 dB M = 16.

At Figure 13, for a higher order modulation, the 16QAM is shown to be the best modulation to obtain high probabilities of low BER. In addition, we can observe that for a BER higher than 10^−2^ the OFDM has the same results as the 16QAM. For a lower BER, the 16QAM and the 16PSK are better. In Figure 13 we can analyze that to obtain a BER lower than 10^−3^ we have a probability of 70% for the 16QAM, a probability of 34% for the 16PSK and a probability of 30% to obtain a BER lower than 2 × 10^−3^ for the OFDM.

For higher BER, to obtain a BER lower than 2 × 10^−2^ we have a probability of 87% to for the OFDM and 16QAM, a probability of 58% for the 16PSK and a probability of 3% for the 16FSK.

### 4.3. BER CDF vs. Power

**Figure 14:**
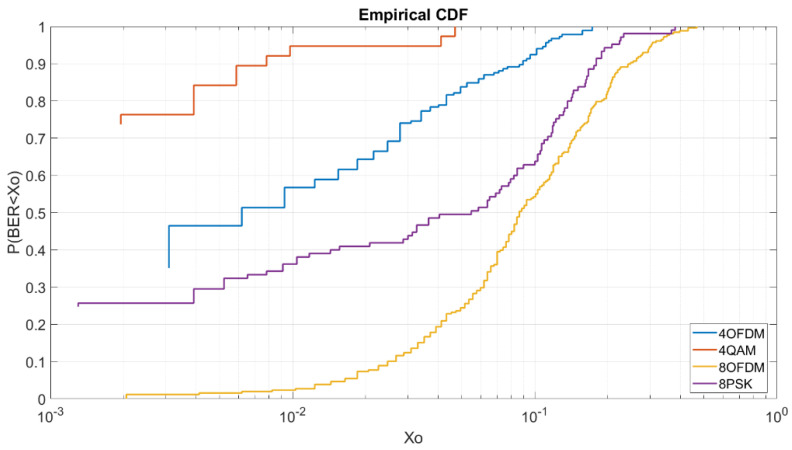
BER CDF vs. Power.

**Figure 15:**
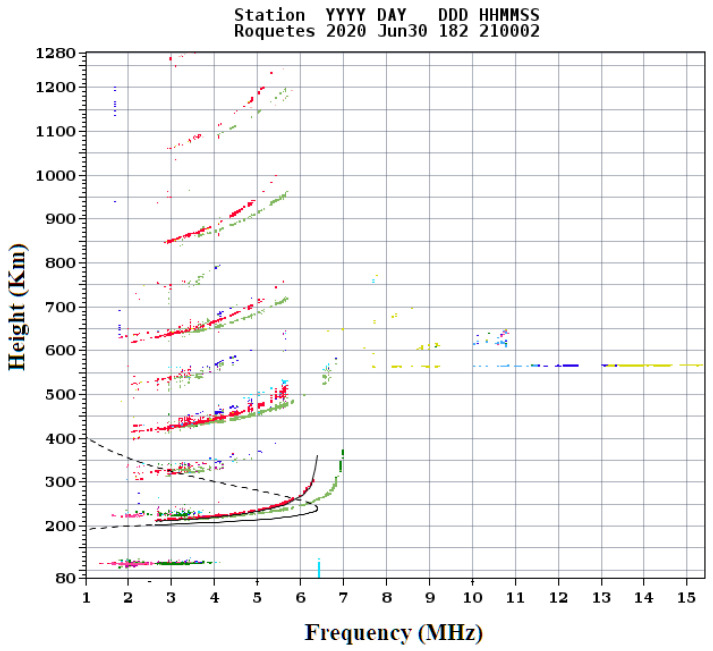
Ionogram at 20 UTC.

Taking into account the results obtained in Section 4.1 BER vs. E_b_/N_0_ and Section 4.2 BER CDF, we can analyze that the most robust modulations for order modulations 4 and 8 are the OFDM with a 4QAM and the OFDM with an 8PSK modulation. Despite the results, during a transmission with an average power, the analysis the E_b_/N_0_ received can have high variations. At Figure 14 we can analyze the CDF BER received signal for low power transmissions of 4QAM, 8PSK, OFDM with 4QAM and OFDM with 8PSK respectively with average power transmissions of 4.7 W, 5.1 W, 3.4 W and 3.7 W. Despite the similar average powers, the efficiency of the OFDM is 63% lower than the narrow-band modulations because of the low value of the IBO. As we can see in Figure 14, the graphic is based on the received signals between 20 UTC and 00 UTC, a range in which the channel presents high delay spreads of 2 ms affecting the robustness of narrowband modulations. To analyze the time range with a greater presence of multipath, we analyzed the ionograms of the Observatori de l’Ebre [22], and in Figure 15 we can distinguish the presence of the different ionosphere layers responsible for signal rebounds. The red line shows the possible reflection of the ordinary wave and the green line shows it for the extraordinary one. In Figure 15 we can see also the multipath produced with more than 8 paths.

At Figure 14, despite the multipath, the 4QAM is the most robust modulation in comparison with the OFDM. As we can see, we have a probability of 76% to receive a BER lower than 2 × 10^−3^, the OFDM with a 4QAM modulation has a probability of 46% to receive a BER lower than 3 × 10^−3^, the 8PSK have a probability 25% to receive a BER lower than 4 × 10^−3^ and the OFDM with an 8PSK has 1% to receive a BER lower than 4 × 10^−3^. As we can see, the OFDM with a 8PSK is shown to be the less robust modulation due to the lack of power to achieve E_b_/N_0_ of 8 dB to maintain its robustness.

### 4.4. Input Back Offs (IBOs) Test

Taking into account the results obtained in Section 4.3 BER CDF vs. Power, the 4QAM was shown to be the most robust modulation followed by the OFDM with a 4QAM modulation. Through the results obtained, the OFDM results can be improved by the variation of the IBO. The OFDM tested in previous figures were configured with an IBO of 3 dB. To improve the robustness of OFDM in front of the 4QAM modulation, in the next sections we will study the best IBO design for the OFDM.

To achieve that goal, a simple IBOs sweep to find the optimal value has been simulated as is shown below. The simulations have been set with a Rayleigh distribution with an SNR of 0 dB and a second path delayed 250 ms from the first one with half of the power. In Table 3, we can see the BER results in terms of IBO in a simulation scenario.

The results show that values of IBOs between around 9 and 10 dBs can reduce the BER around 46% in relation to the IBO of 3 dB, so a priori the increase of the IBO value from the initial one, must overcome the results of the tests already done. That improvement makes it possible to make the OFDM more robust than the 4QAM of Figure 14.

### 4.5. BER CDF vs. IBO

In a real scenario, we performed several tests to verify the simulated IBO results and analyzed the best option to apply. In addition, IoT needs to have a low power system, so we limit the real tests up to 5 W of average power. Different studies such as [28,29,30] show that OFDM IBOs are typical between 6 and 14 dBs. In Table 4, we can see the transmitted PAPR in terms of IBOs and the average power that is transmitted. In Equation (7), we can see how the PAPR is calculated, the maximum absolute voltage value of the signal divided into the average absolute voltage value PAPR is always expressed in dBs.

(7) $$\mathrm{PAPR}\;=\;10\log\left(\frac{\max{\left|x\left(k\right)\right|}^{2}}{E\left[{\left|x\left(k\right)\right|}^{2}\right]}\right)$$

**Figure 16:**
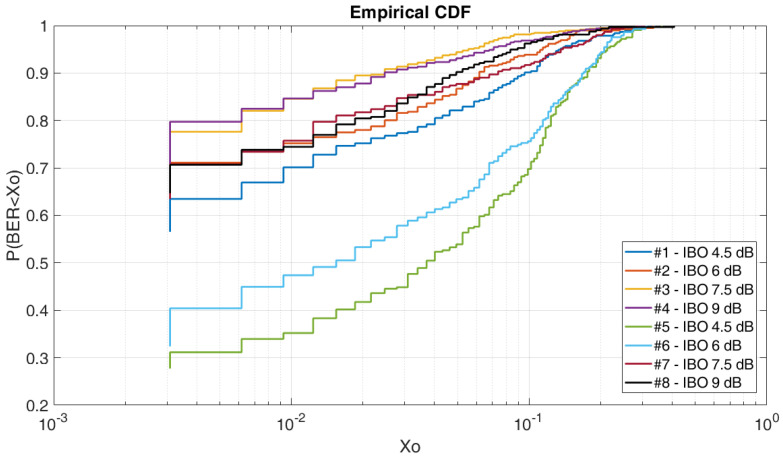
BER CDF vs. IBO.

Through the realization of this test, at Figure 16 we can analyze the IBO tests results of real transmissions. As we can see, to obtain a BER lower than 3 × 10^−3^ we have a probability of 80% for the configuration #4, a probability of a 77% for the configuration #3 and a probability of a 71% for the configuration #2, #8 and #9. The rest of results obtained are lower to assure stable communication, as we can see, to obtain the same BER we have a probability of 63% for the configuration #1, we have a probability of 40% for the configuration #6, and we have a probability of 31% for the configuration #5.

By the variation of the IBO, we can analyze that the OFDM with a 4QAM modulation is more robust than the results achieved with the 4QAM narrowband modulation of Figure 14, the OFDM being more robust for a channel with the presence of a high delay spread.

### 4.6. Single Input Multiple Output (SIMO) Technique

**Figure 17:**
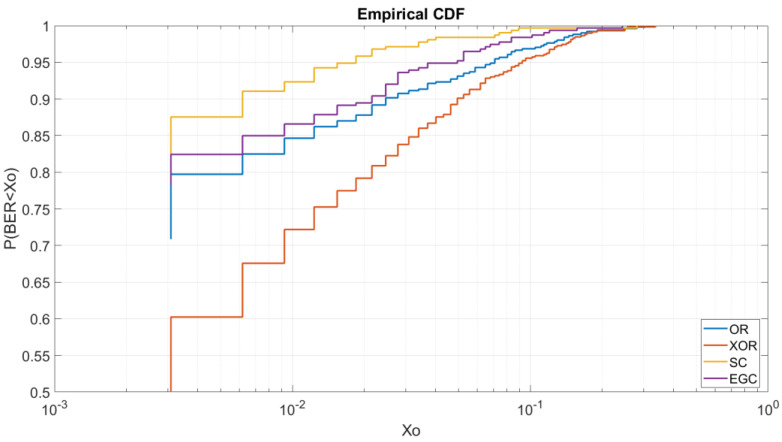
Single input multiple output (SIMO) technique.

Finally, by the results obtained, we can apply SIMO techniques by the addition of a second antenna at the receiver system. In Figure 17, we can see the results obtained by the ordinary wave, the extraordinary wave, and the use of SIMO techniques such as the SC technique and EGC technique. As we can see, the OFDM with a 4QAM modulation, an average power of 4.6 W and a SC technique can improve the probability of receiving a BER lower than 3 × 10^−3^ to a probability of 87% in front of the EGC which improves the probability to receive a BER lower than 3 × 10^−3^ to an 82%.

## 5. Conclusions

Finally, we can conclude that OFDM overcomes narrow-band modulations in scenarios with high multipath such as mornings and evenings for NVIS communications. As we have studied, the OFDM is a good alternative but requires the IBO to be well configured to decrease the PAPR of the modulation signal. This factor is very important for remote sensing where the power consumption is a critical issue.

Through the study performed, in terms of robustness if we analyze the BER E_b_/N_0_ graphics, the OFDM modulation seems to be the best option with an IBO of 3 dB and a modulation order 4 in front of the narrowband modulations, as we have seen in Figure 5. Despite the results, if we focus on a certain value of E_b_/N_0_ and we analyze the BER on a CDF, the results change and the 4QAM seems to be the best option. As analyzed, the 4QAM modulation requires less E_b_/N_0_ to obtain better results as we can see at Section 4.2 BER CDF. That make sense of the challenge to obtain a robust low-power OFDM modulation due to the high PAPR and low average power for the same power transmissions. For that reason, one of the key issues of this study is the optimization of the OFDM using the IBO technique increasing the average power and consequently the average BER as we can see in Figure 16. The IBO technique as we have seen, offers a better efficiency for low-power OFDM modulations for remote sensors.

Furthermore, to make the NVIS communication more robust, as studied, the addition of a second antenna at the receiver system to apply SIMO techniques, can improve the link. By the use of the polarization technique using the ordinary and the extraordinary waves and the SC polarization technique, the BER results have been improved in a 9% the results obtained as we can see at Figure 17.

As analyzed, the mix of the OFDM and QAM depending on the channel scenario is the right approach, especially with the rapid changes of the ionosphere. Through the results obtained, in terms of applications, the OFDM can assure the use of NVIS communication for remote sensors in distances under 250 km, with low-power transmissions and a higher robustness than the HF standards modulations based on narrowband. We conclude that the study of multicarrier modulations benefits the robustness of NVIS communications giving a wide range of possibilities for sensors which need ubiquity to monitoring or sensing multiple facts.

Finally, through the results obtained we propose as the best option for NVIS remote sensing the use of an OFDM modulation for high multipath scenarios with 4QAM subcarriers and an average power transmission of 4.6 W. This configuration offers a bitrate of 2.139 Kbps and a probability of 80% to obtain a BER lower than 3 × 10^−3^. In the case of adding a second antenna in the receiver system, the probability will increase to 87% to obtain a BER lower than 3 × 10^−3^. In the case of need to reduce the average power of the system more than 4.6 W, the receiver node will receive low E_b_/N_0_ signals. In this case, the use of a 4QAM will be more robust than the OFDM modulation.

In Table 5, we summarize different scenarios and the best modulation use for a robust transmission based on the results obtained in this article.

## Figures and Tables

**Table 1:** Orthogonal frequency-division multiplexing (OFDM) configuration for NVIS transmission.

Bandwidth	3 KHz
Useful symbol length	T_S_ = 9.33 ms
Prefix cyclic length	T_CP_ = 3 ms
Number of subcarriers	N_SC_ = 28
Number of pilot OFDM symbols	Ns_P_ = 1
Number of data OFDM symbols	N_D_ = 6
Number of subcarriers DC NULL	N_DC_ = 1
Number of symbols OFDM	N_SOFDM_ = 7
Time duration of OFDM packet	N_PT_ = 86.31 ms
Bits in packet	Bits = 324 bits
Input Back Off	3 dB
Modulation	QPSK
Equalization	Zero forcing
Bitrate of signal frame	2.139 Kbps

**Table 2:** Testbench.

Order of Modulation	Peak Power	Minutes
2, 4, 8, 16, 32	35 dBm	05, 06, 07, 08, 09
2, 4, 8, 16, 32	38 dBm	15, 16, 17, 18, 19
2, 4, 8, 16, 32	41 dBm	25, 26, 27, 28, 29
2, 4, 8, 16, 32	44 dBm	35, 36, 37, 38, 39
2, 4, 8, 16, 32	47 dBm	45, 46, 47, 48, 49
2, 4, 8, 16, 32	50 dBm	55, 56, 57, 58, 59

**Table 3:** Simulated IBO test.

IBO (dBs)	BER
0	0.0863
1.5	0.0644
3	0.0494
4.5	0.0404
6	0.0331
7.5	0.0284
9	0.0273
**10**	**0.0267**
12	0.0295
15	0.0377
18	0.0448
21	0.0497
24	0.0554
27	0.0639

**Table 4:** Real IBO test.

OFDM Configuration	IBO	PAPR	Average Power
#1	4.5 dB	10.3 dB	2.3 W
#2	6 dB	9.1 dB	2.9 W
#3	7.5 dB	8.1 dB	3.7 W
#4	9 dB	7.2 dB	4.6 W
#5	4.5 dB	6.7 dB	2.5 W
#6	6 dB	5.8 dB	3.1 W
#7	7.5 dB	5.0 dB	3.7 W
#8	9 dB	4.4 dB	4.3 W

**Table 5:** Modulation depending on scenario.

Multipath	E_b_/N_0_	Modulation	SIMO Technique	Average Power	PAPR
High	Low	4-QAM	Selection Combining	4.7 W	4.8 dB
High	High	OFDM	Selection Combining	4.6 W	7.2 dB
Low	Low	4-QAM	Selection Combining	4.7 W	4.8 dB
Low	High	OFDM/4-QAM	Selection Combining	4.6W/4.7 W	7.2/4.8 dB
